# Ultrasound Image Quality Comparison Between a Handheld Ultrasound Transducer and Mid-Range Ultrasound Machine

**DOI:** 10.24908/pocus.v7i1.15052

**Published:** 2022-04-21

**Authors:** Nayema Salimi, Antonio Gonzalez-Fiol, N David Yanez, Kristen L Fardelmann, Emily Harmon, Katherine Kohari, Sonya Abdel-Razeq, Urania Magriples, Aymen Alian

**Affiliations:** 1 Department of Anesthesiology, Yale University School of Medicine, Yale New Haven Hospital Connecticut USA; 2 Department of Biostatistics, Yale University School of Public Health, Yale New Haven Hospital Connecticut USA; 3 Integrated Anesthesia Associates, LLC East Hartford, Connecticut USA; 4 Department of Obstetrics, Gynecology and Reproductive Sciences, Yale University School of Medicine, Yale New Haven Hospital Connecticut USA

**Keywords:** handheld ultrasound, obstetrics, image characteristics, obstetric anesthesiology, Butterfly iQ

## Abstract

**Objectives**: Not all labor and delivery floors are equipped with ultrasound machines which can serve the needs of both obstetricians and anesthesiologists. This cross-sectional, blinded, randomized observational study compares the image resolution (RES), detail (DET), and quality (IQ) acquired by a handheld ultrasound, the Butterfly iQ, and a mid-range mobile device, the Sonosite M-turbo US (SU), to evaluate their use as a shared resource. **Methods**: Seventy-four pairs of ultrasound images were obtained for different imaging purposes: 29 for spine (Sp), 15 for transversus abdominis plane (TAP) and 30 for diagnostic obstetrics (OB) purposes. Each location was scanned by both the handheld and mid-range machine, resulting in 148 images. The images were graded by three blinded experienced sonographers on a 10-point Likert scale. **Results**: The mean difference for Sp imaging favored the handheld device (RES: -0.6 [(95% CI -1.1, -0.1), p = 0.017], DET: -0.8 [(95% CI -1.2, -0.3), p = 0.001] and IQ: -0.9 [95% CI-1.3, -0.4, p = 0.001]). For the TAP images, there was no statistical difference in RES or IQ, but DET was favored in the handheld device (-0.8 [(95% CI-1.2, -0.5), p < 0.001]). For OB images, the SU was favored over the handheld device with RES, DET and IQ with mean differences of 1.7 [(95% CI 1.2, 2.1), p < 0.001], 1.6 [(95% CI 1.2, 2.0], p < 0.001] and 1.1 [(95% CI 0.7, 1.5]), p < 0.001), respectively. **Conclusions**: Where resources are limited, a handheld ultrasound may be considered as a potential low-cost alternative to a more expensive ultrasound machine for point of care ultrasonography, better suited to anesthetic vs. diagnostic obstetrical indications.

## Introduction

In a dynamic labor and delivery floor, a handheld/portable device is crucial for assessing fetal heart rate, placental position, and for procedural guidance in a timely and efficient manner. For the obstetrician, the ultrasound has been a vital diagnostic tool since its introduction in 1958 1. More recently, the use of ultrasound (US) technology has gained significant traction in anesthesiology as a clinical and diagnostic tool. However, not all labor and delivery floors are equipped with ultrasound machines which can serve the needs of both obstetricians and anesthesiologists, nor can all afford two different machines to suit their different needs. Limitations to ultrasound use include financial constraints to obtain a device, limited time, limited HIPAA compliant storage space, synchronization with electronic medical records, lack of portability, and steep learning curves for both obtaining and interpreting images 1, 2, 3, 4, 5, 6. Advances in technology have made possible the creation of pocket-sized ultrasound machines that aim to increase ultrasound accessibility by addressing these limitations 1. The increase in accessibility can benefit both patients and trainees as ultrasounds can be utilized in routine care of parturients. Yet, the question remains, does improved portability and access compromise image quality? 

In this study, we evaluate the image characteristics of a handheld device against our standard mobile ultrasound machine. Our study's primary aim was to compare the quality of images obtained by a handheld ultrasound machine (the Butterfly iQ) and our current mobile mid-range ultrasound system, the Sonosite M-turbo US. Given that obstetricians and anesthesiologists routinely use ultrasound, we designed a comparison study utilizing shared resources. This cross-sectional, blinded, and randomized observational study aims to compare the image characteristics acquired by the two ultrasound machines herein described for both obstetric and anesthesiologic purposes. 

## Methods

This prospective observational study was carried out in a tertiary care labor and delivery unit and an outpatient maternal-fetal medicine office. The protocol was approved by the Yale University Institutional Review Board (IRB) and registered on ClinicalTrials.gov (ClinicalTrials.gov Identifier: NCT03764111). The outpatient maternal-fetal medicine office was chosen over the labor and delivery floor and triage due to the differences in acuity to minimize interruptions to patient care and any interference with image acquisition. A total of 75 patients were approached to obtain 30 ultrasound spine (Sp) images, 15 transverse abdominis plane (TAP) images and 30 obstetric (OB) images by each the handheld US (Butterfly iQ; Guilford, CT, USA), and the mid-range Sonosite M Turbo (Bothell, WA, USA). Clinically, the Sp images were obtained to aid with neuraxial anesthesia placement, the TAP images to aid in regional anesthesia nerve blocks for post-cesarean pain, and the OB images for a variety of diagnostic indications, including assessing fetal heart rate, placental position, various measurements of fetal growth, and more.

Pairs of ultrasound images were obtained for the Sp, TAP and diagnostic OB purposes. Each patient was scanned in one of the respective locations by both the handheld and mid-range devices. Images were acquired by two experienced sonologists in their respective fields (AG-F for the Sp and TAP and SA-R for obstetric images). The sonographers were instructed to adjust the gain, depth, and frequency of each probe to optimize the best picture on each machine. 

Spine and TAP images were acquired on the day of a patient’s scheduled cesarean delivery. The 30 obstetric images were obtained as part of the parturients’ prenatal care. All participants agreed to have images taken with both US devices. When utilizing the mid-range US, two types of probes were utilized: a linear array transducer (5 – 11 MHz) for the TAP imaging, and a curvilinear transducer (up to 5 MHz) was utilized for the Sp and OB imaging. On the contrary, the handheld Butterfly iQ relies on capacitive micro-machined ultrasound transducers (CMUTs), allowing for changes in MHz as a preset function (a single probe can scan at different MHz). The images on the handheld Butterfly iQ were performed in the abdomen imaging preset for Sp and OB and on the musculoskeletal preset for TAP images. 

**Figure 1:**
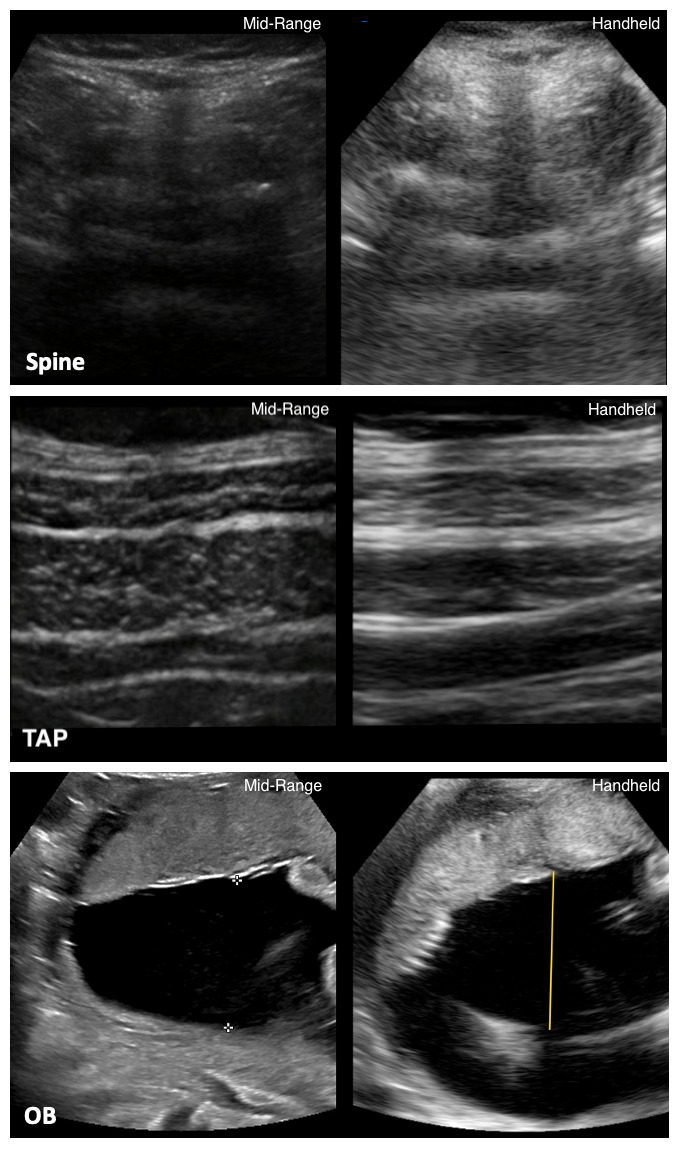
Examples of paired images. Images were unlabeled, cropped, masked, deidentified, and presented in randomized pairs to experienced sonographers for grading. Images were presented by group (Sp, TAP and OB) and graded on a Likert scale from 1-10 on image resolution (RES), detail (DET) and quality (IQ).

Once the images were obtained, they were transferred to a computer, where they were cropped, deidentified, and masked to leave only gray-scale images. The pairs were then randomized for grading (see Figure 1). Three experienced sonologists from each specialty (six raters total) reviewed and rated the images. Sp and TAP images were graded by anesthesiologists familiar with ultrasound use for neuraxial and regional blockade. OB images were graded by experienced physicians from the section of Maternal-Fetal Medicine. Each reviewer rated every pair of images for its resolution (RES), detail (DET), and image quality (IQ).

- RES was defined as the sharpness and crispness of the image and a lack of haziness/blurriness.
- DET was defined as clarity of bone/tissue outlines and ease with which boundaries of structures are seen.
- IQ was an overall assessment encompassing contrast of solid and fluid-filled structures and the absence of noise.

Each of these three qualities was rated using a ten-point Likert scale, as described by Blaivas et al. 7, where 1-3 was defined as “poor”, 4-7 was “good” and 8-10 was “very good” image scores. 

### Statistical Analysis

Our design yielded three rating scores for each image and six rating scores for each patient’s image pairs. We used generalized estimating equation models (GEE) to account for patient-level correlated data to model these data and perform statistical inference. We estimated mean rating scores for RES, DET, and IQ in separate models. We tested for differences in the mean rating scores between the device types using Wald statistics. Hypothesis tests, p-values, and confidence intervals were two-sided. We stratified our analyses by image type: Spine (Sp), the transversus abdominis plane (TAP) and OB images. All analyses were performed with the Stata software package (version 16.1). Measures of inter-rater agreement were computed using the overall percent agreement and intra-rater kappa statistics. The kappa statistics are intra-rater because we computed agreement within rater for the two devices.

## Results

**Figure 2:**
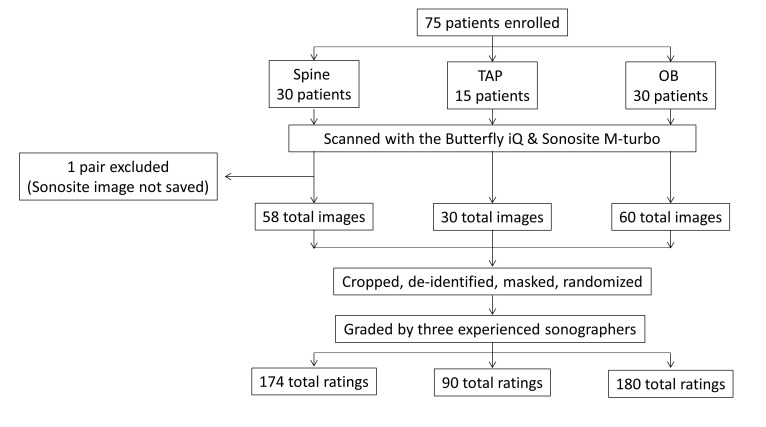
Flow chart of the methods. Patients were enrolled and imaged in one of three groups. Images were obtained by both the handheld Butterfly iQ and mid-range Sonosite M-turbo and the image pairs were cropped, de-identified, masked and randomized before being given to raters. One pair of imagesfrom the Spine group was excluded because the image on the mid-range device was not saved. Total images is the number of images that were graded for each: image resolution, detail, and quality. Graders were experienced sonographers in their respective fields. Spine and TAP blocks were graded by anesthesiologist who routinely use ultrasound in their practice, OB images were graded by experienced obstetricians. TAP = Transversus abdominis plane, OB = obstetric.

A total of 74 image pairs were evaluated by three raters from each specialty: 29 for the Sp, 15 for the TAP, and 30 for OB, for a total of 148 images and 444 ratings for each the handheld and the mid-range US. One of the images from the spine group was not saved to the mid-range device, hence we were unable to compare it (Figure 2). Please see Table 1 for a summary of the mean ratings for Sp, TAP, and OB. Mean differences are with the mid-range US as our reference; positive mean differences indicate that the mid-range unit had a higher rating and negative mean differences indicate that the handheld device had a higher rating (Table 2). 

**Table 1:** Mean ratings of the Sp, TAP and OB images. Ratings were on a ten-point Likert scale, where 1-3 was defined as“poor”, 4-7 was “good” and 8-10 was “very good” image scores.

	**Mid-Range Ultrasound**		**Handheld Ultrasound**	
	Mean	95% CI	Mean	95% CI
**Spine (n = 174)**				
Resolution Detail Quality	6.0 6.0 5.9	5.5 - 6.5 5.5 - 6.4 5.3 - 6.4	6.6 6.8 6.8	6.2 - 7.0 6.4 - 7.1 6.5 - 7.1
**TAP (n = 90)**				
Resolution Detail Quality	5.9 6.0 5.9	5.3 - 6.5 5.5 - 6.5 5.5 - 6.3	6.0 6.8 6.4	5.5 - 6.5 6.5 - 7.1 6.0 - 6.7
**OB (n = 180)**				
Resolution Detail Quality	8.4 8.6 8.2	8.2 - 8.6 8.4 - 8.8 7.9 - 8.4	6.7 7.0 7.1	6.3 - 7.1 6.6 - 7.4 6.7, 7.5

**Table 2:** Mean differences of the Sp, TAP and OB images. Mean differences are with the SU as our reference; positive mean differences indicate that the SU had a higher rating and negative mean differences indicate that the BU had a higher rating.

**Mid-Range vs. Handheld Ultrasound Mean Difference**			
	**Spine **(n = 174)	**TAP **(n = 90)	**OB **(n = 180)
**Resolution**	-0.6 95% CI = (-1.1, -0.1) p = 0.017	-0.1 95% CI = (-1.1, -0.1) p > 0.868	1.7 95% CI (1.2, 2.1) p < 0.001
**Detail**	-0.8 95% CI = (-1.2, -0.3) p = 0.001	-0.8 95% CI = (-1.2, -0.5) p < 0.001	1.6 95% CI = (1.2, 2.0) p < 0.001
**Quality**	-0.9 95% CI = (-1.3, -0.4) p = 0.001	-0.4 95% CI = (-0.9, 0.04) p = 0.072	1.1 95% CI = (0.7, 1.5) p < 0.001

Overall percent agreements were relatively high at 0.71 (0.05); 0.69 (0.05) and 0.67 (0.05) for image RES, DET and IQ. Kappa statistics for RES, DET and IQ were 0.12 (0.09) [95% CI=(-0.06, 0.30)]; 0.10 (0.09) [95% CI=(-0.07, 0.27)] and 0.19 (0.7) [95% CI=(0.04, 0.33)], respectively. 

### Spine

There were 174 rating scores for the spine images for each of the three imaging criteria. Overall, the mean differences in scores for the handheld device and mid-range unit favored the handheld device. Our analyses of the spine sonoanatomy showed a mean RES rating score of 6.6 (95% CI [6.2, 7.0]) for the handheld and a mean score of 6.0 (95% CI [5.5, 6.5]) for the mid-ranged US with a mean difference of -0.6 (95% CI [-1.1, -0.1], p = 0.017). The mean DET score for the handheld was 6.8 (95% CI [6.4, 7.1]), and a mean score for the mid-ranged US images was 6.0 (95% CI [5.5, 6.4]) with a mean difference of -0.8 (95% CI = [-1.2, -0.3], p = 0.001). For the spine total image quality scores, the mean rating score for the handheld device was 6.8 (95% CI [6.5, 7.1]) and 5.9 (95% CI [5.3, 6.4]) for the mid-range US, with a mean difference of -0.9 (95% CI [-1.3, -0.4], p = 0.001). For neuraxial imaging, the image characteristics of the handheld device may be preferable compared to the mid-ranged unit. 

### TAP

There were 90 rating scores for the TAP images for the imaging criteria. Ratings of image characteristics were much more mixed when looking at this image category. The mean RES scores were 6.0 (95% CI = [5.5, 6.5]) for the handheld device and 5.9 (95% CI = [5.3, 6.5]) for the mid-range unit. The difference was -0.1 (95% CI = [-1.1, -0.1]). The size of the mean difference is typical of what one might expect to observe if there was no true difference (p-values > 0.868). The mean DET scores for the TAP images were 6.8 (95% CI = [6.5, 7.1]) for the handheld and 6.0 (95% CI = [5.5, 6.5]) for the mid-range US. The difference in scores was -0.8 (95% CI = [-1.2, -0.5]), indicating the handheld scores were significantly higher (p < 0.001). For the TAP IQ scores, the mean rating for the handheld US was 6.4 (95% CI = [6.0, 6.7]) and 5.9 (95% CI = [5.5, 6.3]) for the mid-range. The difference was -0.4 (95% CI = [-0.9, 0.04]), with a non-statistically significant comparison (p = 0.072). For TAP imaging, there does not seem to be one device that is consistently rated as having better image characteristics. 

### OB

There were 180 rating scores for the OB images each of the three imaging criteria. Unlike the last two image groups, there seemed to be a preference for the mid-range US in these images. The mean RES scores were 6.7 (95% CI = [6.3, 7.1]) for the handheld US and a mean score of 8.4 (95% CI = [8.2, 8.6]) for the mid-range device and a difference of 1.7 (95% CI = [1.2, 2.1], p < 0.001). The mean DET score for the handheld was 7.0 (95% CI = [6.6, 7.4]) and a mean score for the mid-range US images was 8.6 (95% CI = [8.4, 8.8]), with a mean difference of 1.6 (95% CI = [1.2, 2.0], p < 0.001). For the OB IQ scores, the mean rating score for the handheld device was 7.1 (95% CI = [6.7, 7.5]), while the mean score for the mid-range machine was 8.2 (95% CI = [7.9, 8.4]) for a mean difference of 1.1 (95% CI = [0.7, 1.5], p < 0.001). 

## Discussion

Our study was geared towards the assessment and functionality of a handheld ultrasound device that could be shared amongst both obstetricians and anesthesiologists in a dynamic labor and delivery floor. The main outcome of this study was to compare the image characteristics of these devices, focusing on image RES, DET and IQ. 

The obstetric providers preferred the mid-range machine over the handheld device. The results for the handheld device mostly on the high spectrum of a “good image” (4-7 score) while the mid-range unit scored on the “very good image” (8-10) range. That is, the obstetric images for both devices were rated mainly between the 7-8.6, which accounts for good and very good images. Overall percentage agreement and Kappa statistics show good agreement between and amongst raters. When evaluating the anesthesia-related imaging, our study showed that the handheld device provided better RES, DET, and IQ when evaluating neuraxial or Sp imaging than the mid-range device. Similarly, when comparing the TAP block images, there was a tendency towards better RES, DET and IQ from the handheld device. Yet, the only one that achieved statistical significance was the detail of the image. 

There are several plausible explanations as to why there was a difference in the rating scores of the obstetricians and anesthesiologists. One may have to do with the ultrasound technology used in each of the respective ultrasounds. As described earlier, traditional US technology, as in the mid-range US, depends on ultrasound waves emitted from piezoelectric crystals, while new technology in the handheld device utilizes CMUTs for this purpose 8, 9. There may be a difference in how these technologies produce images on different wave-structure interfaces (e.g., bone, soft tissue, or fascial planes). Additionally, obstetricians, more than anesthesiologists, are trained by evaluating images from high end consoles and are therefore conditioned to notice even small differences in image quality. Despite the differences, all evaluators agreed that the images from both devices were good, and sufficient for performing routine bedside scans in the maternity ward. The small difference in scores should be accounted for when considering the 20 times price difference between devices ($2,000 vs. $40,000). The addition of handheld devices at our institution has increased the availability of ultrasound from 2 to 6 devices with a moderate investment. 

Increased availability of a handheld device may improve both faculty and trainees' scanning skills. Ultrasonography skill acquisition and retention require practice and constant feedback given that imaging is very operator dependent 3, 6. Some authors have proposed that the number of examinations and competence may not be linearly correlated 6. Both the obstetric and anesthesiology literature coincides with the need for more hands-on US time and curriculum that address its use and correct interpretation 6, 10, 11. For programs to be able to provide such a curriculum, more ultrasound devices are needed in the hands of trainees with live feedback readily relayed. Independent of the technology utilized, the image should be reliable, and the US should be affordable and portable. A handheld device improves the availability to quickly deploy resources to the needed location without carrying cumbersome heavy equipment that requires draping or disinfection after each patient use. In our study, the handheld US weighs in at 0.69 lbs vs. 6.7 lbs for our mid-range unit– not including traveling cart. Smaller size may especially be of use when evaluating parturients outside of the labor and delivery floor as well, such as in the emergency department or perioperative areas for fetal heart rate.

At our institution, the increased availability of US devices has improved the hands-on experience for trainees and increased the frequency at which ultrasound is used. Although not evaluated in our study, the increased availability and trainee’s ability to share de-identified images via encrypted emails, increases the amount of images available for review and improves the feedback they receive. The ability of providers to remotely review an image could not only help to facilitate and expedite care for their patients, but also to increase ad hoc teaching opportunities. 

One of this study's strengths is the number of images reviewed by three graders from each specialty. Additionally, we compared the capabilities of the handheld Butterfly iQ vs. our current mid-ranged US by testing both the linear and curvilinear presets. We considered this an important addition since most obstetric anesthesiology divisions with financial restrictions would use labor and delivery resources. In general, this means that anesthesiologists would have access to a curvilinear probe, but not a linear probe. Linear probes are essential for anesthesiologists to perform US-guided intravenous, arterial insertions, central line insertions, and transversus abdominis plane (TAP) blocks. There is evidence that the use of liposomal bupivacaine for TAP block for post-cesarean pain may improve patient satisfaction and overall narcotic consumption and having an accessible linear probe for providing this procedure seemed prudent 12, 13, 14. Another advantage of our study is that we did not rely on volunteers; rather, we recruited patients with various body mass indexes. 

One of our study's limitations was that the handheld US images' acquisition was directly acquired from the Butterfly network cloud, whereas the imaging from our current US was extracted from the machine hard drive and then imported into a PowerPoint presentation. The latter could have resulted in the degradation of images during the transfer as described by Blaivas et al. 15. Since the images from the handheld device were already in a digital format, they may have been affected the least by the transfer. 

## Conclusions

When comparing ultrasounds on image characteristics alone, the handheld US was rated lower when used for obstetrical purposes. However, RES, DET and IQ of the handheld device was still rated as being “good”. The ideal ultrasound in the inpatient setting should be affordable and portable while maintaining comparable image quality to high-end ultrasound machines 15, 16. Secondary to advancements in technology, both the cost and portability (size) of US machines have been reduced over the last decade. A handheld ultrasound may be considered as a potential low-cost alternative to a more expensive ultrasound machine for point of care ultrasonography, better suited to anesthetic vs. diagnostic obstetrical indications. 

## Statement of Ethics Approval 

The protocol was approved by the Yale University Institutional Review Board (IRB) and registered on ClinicalTrials.gov (ClinicalTrials.gov Identifier: NCT03764111).

## Disclosures

None.
